# Multi-Omics Analysis Identifies the Key Defence Pathways in Chinese Cabbage Responding to Black Spot Disease

**DOI:** 10.3390/genes17010115

**Published:** 2026-01-21

**Authors:** Wenyuan Yan, Hong Zhang, Weiqiang Fan, Xiaohui Liu, Zhiyin Huang, Yong Wang, Yerong Zhu, Chaonan Wang, Bin Zhang

**Affiliations:** 1 College of Life Sciences, Nankai University, Weijin Road 94, Tianjin 300071, China; 2 Tianjin Academy of Agricultural Sciences, Vegetable Research Institute, Tianjin 300381, China; 3 State Key Laboratory of Vegetable Biobreeding, Tianjin Academy of Agricultural Sciences, Tianjin 300192, China; 4 Tianjin Kernel Agricultural Science and Technology Co., Ltd., Vegetable Research Institute, Tianjin 300381, China

**Keywords:** *Alternaria brassicicola*, *BraPBL*, Chinese cabbage, metabolome, transcriptome

## Abstract

Background: Black spot disease severely constrains Chinese cabbage production. Methods: To elucidate the defence mechanisms underlying this response, transcriptomic and metabolomic profiles were analysed in leaves of the Chinese cabbage line 904B at 24 h post-inoculation (hpi) with *Alternaria brassicicola*. In parallel, gene silencing and overexpression were conducted for *BraPBL*, an RLCK family member in Chinese cabbage. Results: The Chinese cabbage line 904B exhibited marked suppression of cytokinin and auxin signalling, coupled with enhanced expression of genes involved in ethylene and jasmonic acid signalling. Multiple secondary metabolites exhibited differential changes, specifically the sterol compound 4,4-dimethyl-5alpha-cholest-7-en-3beta-ol was significantly upregulated in the treatment group. These metabolites were primarily enriched in the indole alkaloid metabolism and glycerolipid metabolism pathways. Concurrently, *BraPBL* exhibits increasing expression with prolonged infection. *BraPBL* overexpression enhances resistance to black spot disease, whereas silencing reduces resistance. Subcellular localization confirmed BraPBL at the plasma membrane. Overexpression of *BraPBL* upregulates the reactive oxygen species-related gene *RBOH* and the signal transduction-related gene *MEKK1*, whilst simultaneously activating the JA pathway. Conclusions: Overall, 904B activates defence-related hormones while suppressing growth and development-related hormones during early infection. Secondary metabolites, particularly the sterol compound 4,4-dimethyl-5alpha-cholest-7-en-3beta-ol, play key roles in defence, and *BraPBL* functions as a black spot disease–related defence gene in Chinese cabbage.

## 1. Introduction

Chinese cabbage (*Brassica rapa* L. ssp. *pekinensis*) is a widely cultivated vegetable valued for its high nutritional content, productivity, and storability [1]. In 2023, global cabbage production amounted to 73,828,504 tonnes, with Chinese cabbage accounting for a significant proportion thereof (FAOSTAT data). However, its cultivation is frequently disrupted by black spot disease, also referred to as Alternaria blight disease, predominantly caused by *Alternaria brassicae* and *A. brassicicola* [2,3,4]. These pathogens infect plants throughout the entire growth cycle, leading to tissue decay and substantial yield and quality losses [5]. Infected areas develop brownish-black concentric rings, often accompanied by a pale-yellow halo of chlorosis. Under favourable conditions, a layer of brownish-black conidia and distinct necrotic zones form at the lesion site [6]. In the margins of the chlorotic and necrotic zones, mesophyll cells—particularly those adjacent to the hyphae—exhibit varying degrees of plasmolysis, disintegration of organelles, and swelling of the nuclear membrane. Concurrently, these cells contain numerous lytic vacuoles, membranous multilamellar structures, and vesicular bodies. Within the chloroplast, the chloroplast envelope degenerates, grana disappear, the thylakoid lumen expands, and the stroma disintegrates [7]. Through infection of seeds and seedlings, causing losses of 15% to 70% [8]. Black spot disease is particularly prevalent in tropical and subtropical regions, and environments characterised by high humidity and frequent rainfall also promote its occurrence [9].

Through long-term co-evolution with pathogens, plants have developed a two-layer immune system: pathogen-associated molecular pattern–triggered immunity (PAMP-triggered immunity, PTI) and effector-triggered immunity (ETI) [10]. ETI exhibits a stronger and more persistent defence response than PTI [11]. During the ETI process, the infected site undergoes a localised hypersensitive response (HR) of cellular necrosis, thereby resisting pathogen invasion and preventing further spread [12,13]. This localised HR signal can trigger the expression of defence genes throughout the entire plant, subsequently inducing broad-spectrum resistance against pathogens across the whole plant, that is, systemic acquired resistance. These defence responses are mediated by diverse signalling components, including mitogen-activated protein kinases (MAPKs), receptor-like cytoplasmic kinases (RLCKs), reactive oxygen species (ROS), jasmonic acid (JA), salicylic acid (SA), ethylene (ET), and secondary metabolites [14,15].

SA, JA, and ET play crucial roles in plant-pathogen interactions [16]. In *Arabidopsis thaliana*, JA enhances resistance to necrotrophic pathogens; conversely, susceptibility to biotrophic pathogens is increased. ET is a vital gaseous hormone within plants, primarily regulating processes such as seed germination and growth, leaf and tissue senescence, and fruit ripening. It also plays a significant role in the response to both biotic and abiotic stresses [17]. Extensive research indicates that ET participates in regulating the immune responses of various plants, including Arabidopsis, tobacco, tomato, rice, and soybean. In plant immune responses, ET is generally considered to act synergistically with JA in mediating resistance to necrotrophic pathogens, whilst antagonising SA-mediated resistance to biotrophic pathogens [18].

Secondary metabolites are a diverse array of structurally distinct small-molecule organic compounds produced by plant secondary metabolism. They are extensively involved in biochemical processes such as plant pigment synthesis, growth and development, signal transduction, antioxidant mechanisms, and stress defence. Phytosterols are essential components of cell membranes, playing a crucial role in various physiological and biochemical processes during plant development and stress resistance [19]. Phytosterols help maintain the integrity, fluidity, and permeability of the membrane lipid bilayer, thereby enhancing the stress resistance of plants [20].

Plants primarily perceive extracellular signalling molecules of various natures through surface receptors on the cell membrane, such as receptor-like kinases (RLKs) and receptor-like proteins (RLPs) [21,22]. Large portions of the RLKs family lack extracellular domains; these members are termed receptor-like cytoplasmic kinases (RLCKs). Many RLCKs are localised to the plasma membrane via N-myristoylation or palmitoylation [23]. The majority of RLCKs contain only a single serine/threonine kinase domain, whilst a subset of RLCKs additionally harbour LRR, EGF, WD40, or transmembrane domains [24,25]. RLCKs are typically coupled with RLKs/RLPs to mediate signal transduction, transmitting cellular signals through the phosphorylation of downstream components, thereby regulating various physiological responses [26]. RLCKs function as key downstream signalling hubs for multiple pattern-recognition receptors (PRRs), and they are integral to plant responses to pathogens and abiotic stresses [27,28,29]. The early defence responses of Chinese cabbage against *A. brassicicola*, as well as the role of RLCK family members in the resistance to black spot disease, are yet to be fully elucidated. Recently, integrated transcriptomic and metabolomic analyses have been extensively employed to identify candidate genes and metabolites playing crucial roles in stress responses and quality-related traits. To characterise the early defence mechanism of Chinese cabbage against *A. brassicicola*, this study examined differentially expressed genes and metabolites at 24 hpi and assessed the functional relevance of the RLCK family member *BraPBL* through gene silencing and overexpression. These findings provide foundational insights into the defence strategies of Chinese cabbage and offer useful references for breeding programmes aimed at enhancing resistance to black spot disease.

## 2. Materials and Methods

### 2.1. Materials and Experimental Treatment

Leaves from the Chinese cabbage inbred line 904B (A disease-resistant variety of Qingma leaf-type Chinese cabbage) were inoculated with *A. brassicicola* and sampled at 24 h post-inoculation (hpi). Control plants were treated with sterile water and sampled at the same time points. Each treatment included three biological replicates. All samples were flash-frozen in liquid nitrogen and stored at −80 °C for transcriptomic and metabolomic analyses.

### 2.2. Pathogen Inoculation and Disease Scoring

A 10 µL droplet of *A. brassicicola* spore suspension (1 × 10^5^ spores/mL) was applied to each leaf, which was then covered with plastic wrap and incubated at 25 °C and 96% relative humidity under alternating dark/light periods (A 24 h period of darkness serves as the pre-treatment prior to inoculation, followed by 8 h of light and 16 h of dark). Disease severity was assessed using a six-level scale: 0 (no symptoms), 1 (<5% lesion area), 3 (6–10%), 5 (11–20%), 7 (21–50%), and 9 (>51%) [30].

### 2.3. RNA Sequencing

Total RNA was extracted using TRIzol (Invitrogen, Carlsbad, CA, USA), treated with TURBO DNase I (Ambion, Austin, TX, USA), and purified with an RNeasy^®^ Plant Mini Kit (QIAGEN, Hilden, Germany). Libraries were prepared with a TruSeq RNA Sample Prep V2 Kit (Illumina, San Diego, CA, USA). Library quality was assessed using an Agilent 2200 TapeStation (Agilent, Santa Clara, CA, USA), and sequencing was performed on an Illumina NovaSeq 6000 platform using 150 bp paired-end reads. Sequencing data are available in the NCBI database (SRR28286398, SRR28286399, SRR28286400, SRR28286402, SRR28286392, SRR28286391).

### 2.4. Transcriptomic Data Analysis

The *Brassica rapa* reference genome (Brara_Chiifu_V3.5) and annotations were downloaded from the Brassicaceae Database (http://www.brassicadb.cn/#/Download/, accessed on 7 November 2023). Raw reads were filtered with Fastp V0.23.4 and aligned to the genome using Hisat2 V2.1.0 [31,32]. Gene expression levels were quantified as fragments per kilobase of transcript per million mapped reads (FPKM) using StringTie V2.2.3 [33]. Differentially expressed genes (DEGs) were identified using DESeq2 V1.30.0 with thresholds of |log_2_(fold change)| ≥ 1 and FDR < 0.05 [34]. Identification of transcription factors and resistance genes based on annotations from the Brassicaceae Database. AgriGO (http://systemsbiology.cau.edu.cn/agriGOv2/#, accessed on 7 November 2023) was employed for Gene Ontology (GO) term enrichment analysis. Metabolic pathway analysis was performed using KEGG (https://www.kegg.jp/, accessed on 7 November 2023).

### 2.5. Metabolite Extraction and Data Analysis

Metabolite extraction and LC–MS/MS followed previously described procedures [30]. Raw MassLynx V4.2 data were processed in Progenesis QI V2.2 for peak picking, alignment, and compound identification using the METLIN database, with theoretical fragment matching and mass deviation < 100 ppm. Peak areas were normalised to total ion intensity prior to analysis. Identified metabolites were annotated using KEGG (https://www.kegg.jp/, accessed on 7 November 2023), HMDB (https://hmdb.ca/, accessed on 7 November 2023), and LIPID MAPS (https://www.lipidmaps.org/, accessed on 7 November 2023). OPLS-DA modelling was performed using ROPLS V1.6.2, and differentially accumulated metabolites (DAMs) were defined by FC > 1, *p* < 0.05, and VIP > 1 [35,36].

### 2.6. RNA Extraction and qRT-PCR

Total RNA was extracted using RNAiso Plus (TaKaRa, Kyoto, Japan), and reverse transcription was conducted with the TaqMan Reverse Transcription Reagent (Applied Biosystems, Foster, CA, USA). qRT-PCR was performed using SYBR Premix Ex Taq™ II (TaKaRa, Kyoto, Japan) with *EF-1α* as the internal reference. Relative expression levels were calculated using the 2^−∆∆Ct^ method [37]. All qRT-PCR reactions were conducted in technical triplicate. Primer sequences are listed in the Supplementary Table S1.

### 2.7. VIGS-Mediated Silencing of BraPBL

A *BraPBL*-specific fragment was selected using the Sol Genomics Network VIGS tool (https://vigs.solgenomics.net/, accessed on 17 February 2025) and inserted into the pTRV2 vector to generate pTRV2-BraPBL. Empty pTRV1/pTRV2 constructs served as negative controls. *Agrobacterium* cultures carrying the constructs were resuspended to OD_600_ = 1.0 in infiltration buffer (10 mM MgCl_2_, 200 mM AS, 10 mM MES, pH 5.6). Seedlings (*Brassica rapa* cv. J405) were vacuum infiltrated at −0.05 MPa for 5 min, repeated twice, then incubated in darkness at 20 °C for 48 h before transplantation for subsequent analyses [30].

### 2.8. Generation of BraPBL Overexpressing Plants

The *BraPBL* coding sequence was amplified and cloned into the 35S overexpression vector. Constructs were introduced into *Agrobacterium tumefaciens* GV3101. Chinese cabbage (*Brassica rapa* cv. 49caixin) transformation followed previously described *Agrobacterium*-mediated protocols [38]. Chinese cabbage seeds were disinfected with 75% ethanol for 30 s and 10% sodium hypochlorite for 1 min, followed by 3 rinses with sterile water. The sterilised seeds were sown onto germination medium (4.43 g/L Murashige and Skoog Basal Medium with Vitamins M519, 20 g/L sucrose, 8 g/L agar, pH 5.8). Maintain the tissue culture at 25 °C under 12 h of light per day. Cotyledons (with petioles) were excised from 4-day-old seedlings and immediately inoculated with *A*. *tumefaciens* GV3101 (OD600 = 0.2) for 1 min. The infected explants were transferred to co-culture medium (4.43 g/L M519, 20 g/L sucrose, 8 g/L agar, 2 mg/L 6-benzylaminopurine, 1 mg/L 1-naphthaleneacetic acid, 7.5 mg/L AgNO_3_, pH 5.8) and cultured in the dark for 2 days. The explants were then transferred to selection medium (4.43 g/L M519, 20 g/L sucrose, 8 g/L agar, 2 mg/L 6-benzylaminopurine, 1 mg/L 1-naphthaleneacetic acid, 7.5 mg/L AgNO_3_, 50 mg/L kanamycin, 250 mg/L carbenicillin, pH 5.8) and cultured under standard conditions for 4 weeks. Once the explants differentiate into buds reaching 2 cm in length, they are transferred to subculture medium (4.43 g/L M519, 20 g/L sucrose, 8 g/L agar, 2 mg/L 6-benzylaminopurine, 1 mg/L 1-naphthaleneacetic acid, 7.5 mg/L AgNO_3_, 25 mg/L kanamycin, 250 mg/L carbenicillin, pH 5.8). When the seedlings reach 4 cm in height, transfer them to rooting medium (4.43 g/L M519, 20 g/L sucrose, 8 g/L agar, 1 mg/L 1-naphthaleneacetic acid, pH 5.8).

### 2.9. Subcellular Co-Localization

*BraPBL* coding sequences (without stop codon) were fused to GFP in the pCAMBIA1304 vector. Constructs (35S:BraPBL-GFP and 35S:GFP control) were co-infiltrated with 35S:OsMCA1-RFP (plasma membrane marker) into tobacco leaves via *A. tumefaciens* GV3101. Fluorescence was observed under a Leica TCS SP8 confocal microscope three days post-infiltration [39].

### 2.10. NBT Staining

Leaves were incubated in a 0.1% NBT solution in darkness for 5 h at room temperature, followed by incubation in anhydrous ethanol at 65 °C for 30 min, until they became decolorized and transparent. The experiment employed three independent biological replicates.

### 2.11. Trypan Blue Staining

Leaves were immersed in 0.4% trypan blue, boiled for 3 min, and incubated overnight at room temperature. Tissues were decolorized in 75% ethanol three times for 30 min each and subsequently imaged after final immersion in 95% ethanol. The experiment employed three independent biological replicates.

## 3. Results

### 3.1. Differentially Expressed Genes in Chinese Cabbage Responding to Black Spot Disease

**Figure 1:**
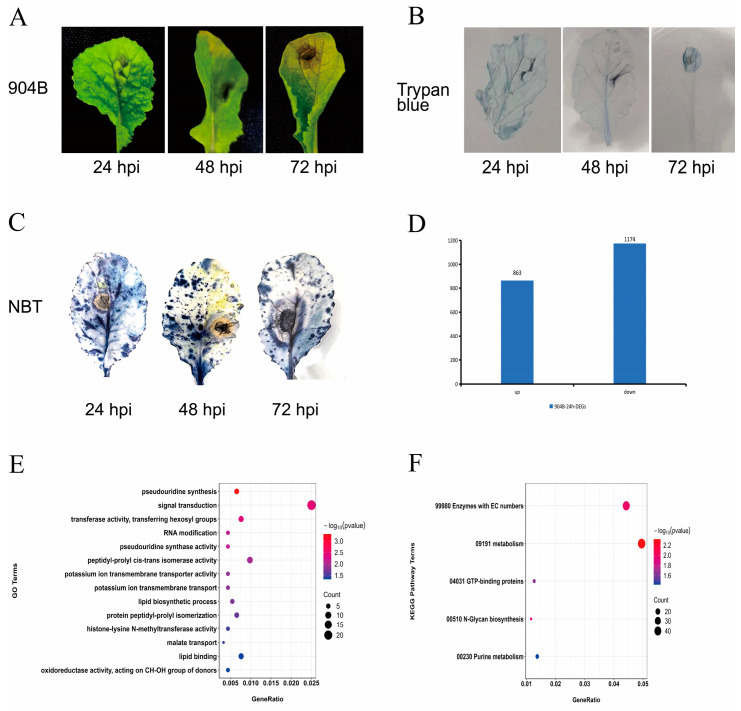
Analysis of DEGs in 904B genotype Chinese cabbage in response to black spot disease. (**A**) The disease symptoms of Chinese cabbage leaves at different time points following inoculation with *A. brassicicola*; (**B**) The trypan blue staining of Chinese cabbage leaves at different time points following inoculation with *A. brassicicola*; (**C**) The NBT staining of Chinese cabbage leaves at different time points following inoculation with *A. brassicicola*; (**D**) Number of DEGs in 904B at 24 hpi; (**E**) GO enrichment of DEGs at 24 hpi; (**F**) KEGG enrichment of DEGs at 24 hpi.

At 24 hpi, no visible symptoms appeared on the leaves of Chinese cabbage line 904B, without staining. However, following NBT staining, brown spots emerged at the inoculation sites, surrounded by a blue halo. The area of the spot expanded as the infection progressed. Trypan blue specifically stains dead cells blue, while living cells remain unstained. The trypan blue staining reveals a blue necrotic zone surrounding the inoculation site at 48 hpi. The area of brown spots and necrotic regions expanded further, with a pale-yellow halo appearing around the spot at 72 hpi (Figure 1A–C).

To further explore the early molecular response of Chinese cabbage to *A. brassicicola*, transcriptome sequencing was performed on infected and control leaves at 24 hpi (Supplementary Table S2). A total of 2037 DEGs were obtained, including 863 up-regulated genes and 1174 down-regulated genes (Figure 1D). RT-qPCR validation of DEGs yielded an R^2^ of 0.92 (Supplementary Figure S1). GO enrichment analysis revealed that DEGs were mainly involved in signal transduction, lipid biosynthetic process, and oxidoreductase activity. KEGG enrichment analysis showed that DEGs were mainly associated with metabolism, GTP-binding protein, and N-Glycan biosynthesis pathways (Figure 1E,F).

### 3.2. Responses of Hormone-Signalling and Defence-Related Genes in Chinese Cabbage to Black Spot Disease

**Figure 2:**
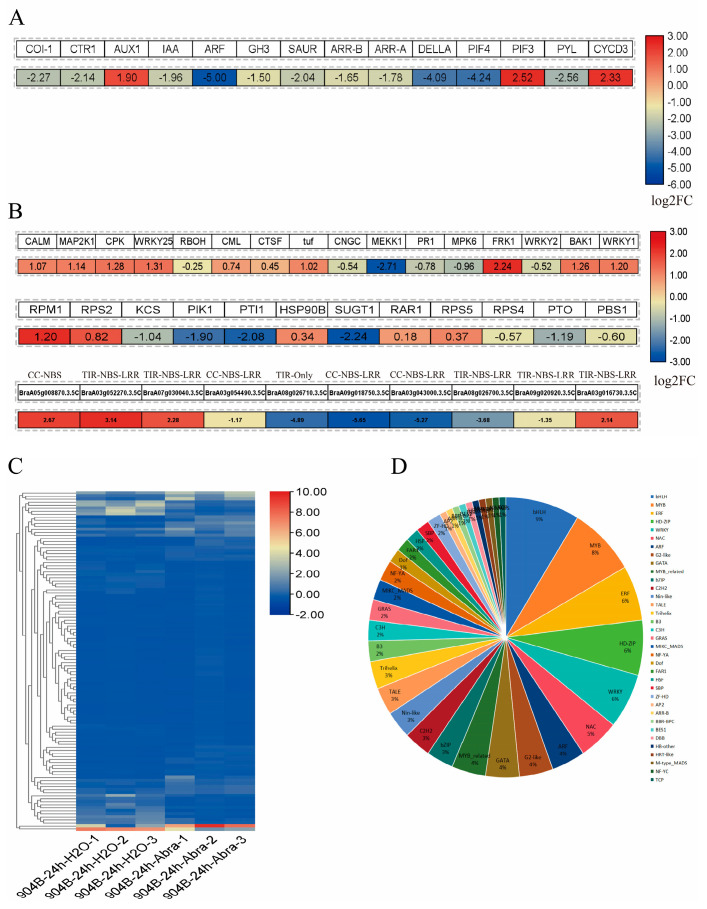
Expression of genes associated with hormone signalling and disease defence pathways in Chinese cabbage line 904B at 24 hpi. (**A**) Expression of hormone-related differentially expressed genes; (**B**) Expression of disease defence-related differentially expressed genes; (**C**) Transcription factors expression heatmap; (**D**) Transcription factors distribution proportion.

Genes involved in auxin, cytokinin, gibberellin, abscisic acid, and brassinosteroid signalling displayed marked expression changes following infection. The gene *COI-1* in the JA pathway and the gene *CTR1* in the ET pathway were both significantly downregulated. A total of 5 DEGs were detected in the auxin pathway, with only *AUX1* exhibiting upregulation, while the remaining genes all showed downregulation. Both key genes *ARR-A* and *ARR-B* in the cytokinin pathway were downregulated. Additionally, it was found that the gene *PIF3* in the gibberellin pathway was upregulated, while the other 2 genes were downregulated. The *PYL* gene was downregulated in the abscisic acid pathway. The *CYCD3* gene was upregulated in the brassinosteroid pathway (Figure 2A).

Defence-related pathways were also induced. In the PTI pathway, genes such as *CALM*, *CPK*, and *MAP2K1* were significantly upregulated. Genes such as *RPM1*, *RPS2*, *RPS5*, and *RAR1*, in the ETI pathway, showed significantly upregulated expression. Simultaneously, multiple R genes possessing conserved disease resistance domains were observed to be activated (Figure 2B).

Transcription factors play a crucial role in plant growth, development, and stress responses. Following infection, a substantial number of transcription factors were found to be expressed in Chinese cabbage (Figure 2C, Supplementary Table S3). These transcription factors can be classified into 35 categories, with the *BHLH* class accounting for the highest proportion (Figure 2D).

### 3.3. Differentially Expressed Metabolites in Chinese Cabbage Responding to Black Spot Disease

**Figure 3:**
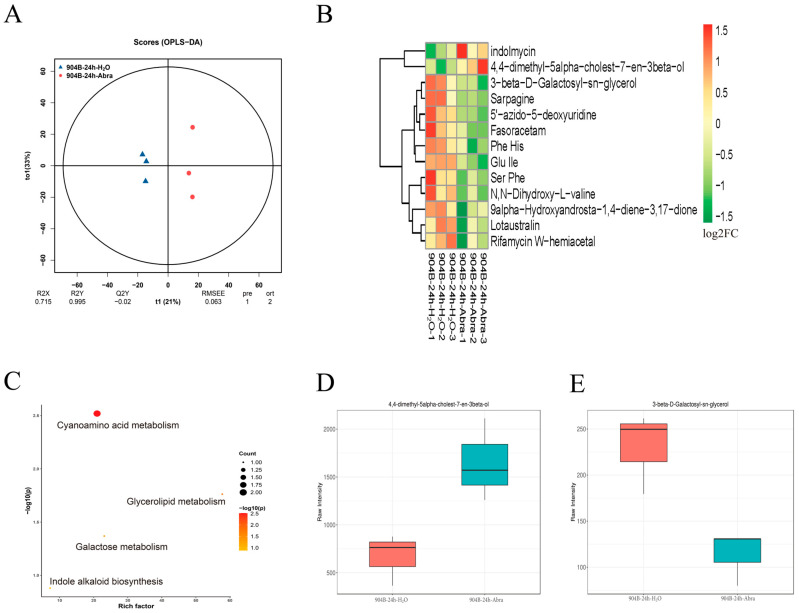
Differentially expressed metabolites in 904B Chinese cabbage at 24 hpi. (**A**) OPLS plot of metabolites across different treatment groups; (**B**) Expression heatmap of differentially expressed metabolites; (**C**) KEGG annotations of differentially expressed metabolites; (**D**) Raw intensity of 4,4-dimethyl-5alpha-cholest-7-en-3beta-ol in different treatment groups; (**E**) Raw intensity of 3-beta-D-galactosyl-sn-glycerol in different treatment groups.

To complement the transcriptomic findings, non-targeted metabolomics was performed on infected and control leaves at 24 hpi. Across datasets, 9453 peaks were detected and annotated to 2621 metabolites, including 6246 peaks (1436 metabolites) in positive ion mode and 3207 peaks (1185 metabolites) in negative ion mode (Supplementary Table S4). Metabolites from different samples within the same treatment group were clustered effectively into a single category (Figure 3A). Among these, metabolites such as N,N-dihydroxy-L-valine exhibited significantly differential expression (Figure 3B). KEGG enrichment analysis indicated that differentially expressed metabolites at 24 hpi were mainly associated with indole alkaloid biosynthesis metabolism and glycerolipid metabolism pathway (Figure 3C). In particular, the sterol compound 4,4-dimethyl-5alpha-cholest-7-en-3beta-ol exhibited significantly upregulated expression in the treatment group, whereas the glycosylglycerol ester compound 3-beta-D-galactosyl-sn-glycerol showed significantly downregulated expression (Figure 3D,E).

### 3.4. Silencing BraPBL Compromises Resistance in Chinese Cabbage

**Figure 4:**
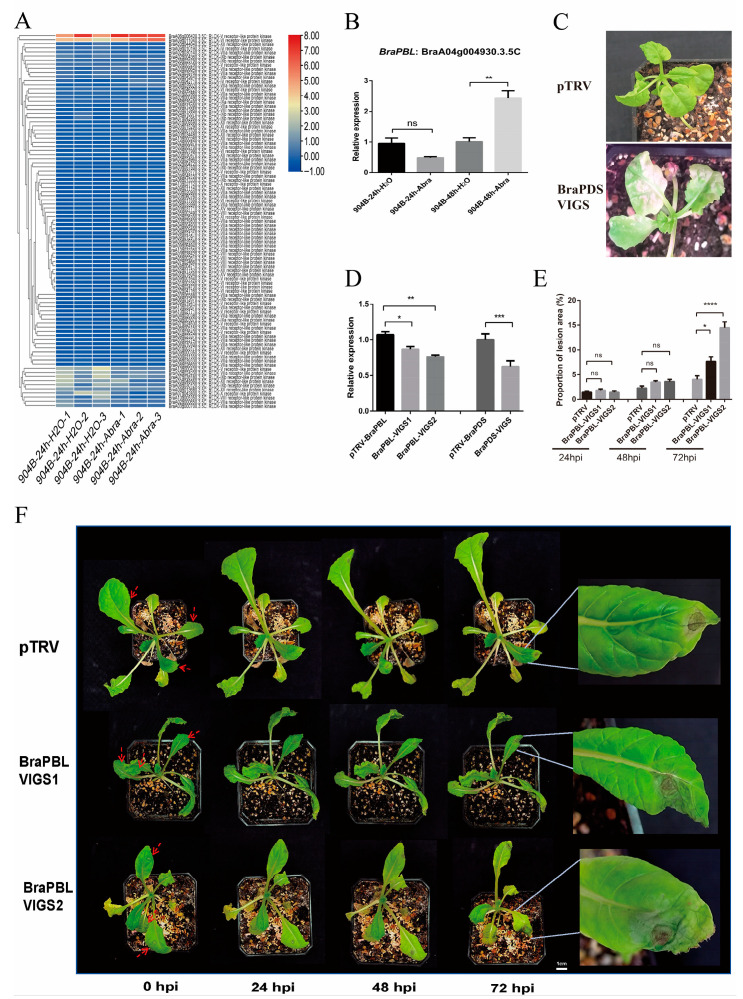
Silencing of *BraPBL* in Chinese cabbage via VIGS. (**A**) Expression of RLCK family members in 904B at 24 hpi; (**B**) Relative *BraPBL* expression in 904B; (**C**) Phenotype of *BraPDS*-silenced Chinese cabbage (pTRV = control); (**D**) Relative expression in *BraPBL*-silenced plants; pTRV-*BraPBL* and pTRV-*BraPDS* served as controls; (**E**) Percentage of lesion area in different treatment groups; (**F**) Representative disease symptoms in different treatment groups. Red arrows mark inoculation sites. Bar = 1 cm. ****, ***, **, *, and ns indicate *p* < 0.0001, *p* < 0.001, *p* < 0.01, *p* < 0.05, and not significant, respectively. Data are presented as means ± standard deviation from three independent biological replicates.

Given the established role of RLCKs in pathogen-responsive signalling, especially *PBL*, the expression of 91 RLCK family members was examined (Figure 4A). Chinese cabbage line 904B showed minimal early response but strong upregulation at 48 hpi (Figure 4B), suggesting a potential link between *BraPBL* activation and enhanced defence.

To test this hypothesis, *BraPBL* was silenced using VIGS and confirmed through PCR and expression analysis (Supplementary Figure S2). The *PDS* gene in Chinese cabbage was silenced, resulting in a white leaf phenotype (Figure 4C), alongside reduced *PDS* gene expression levels (Figure 4D). This demonstrates the reliability of the silencing system. Pathogen inoculation assays were then performed. At 24 and 48 hpi, lesion areas did not differ significantly between BraPBL-VIGS and control plants, and all samples remained at disease severity level 1. By 72 hpi, however, BraPBL-VIGS plants exhibited markedly larger lesions (Figure 4E). Control plants remained at severity level 1, showing small brown lesions with a yellow halo. In contrast, BraPBL-VIGS1 plants reached severity level 3, developing brown concentric rings with limited fungal growth, while BraPBL-VIGS2 plants reached severity level 5, displaying larger concentric rings, sparse fungal growth, and clear necrotic zones (Figure 4F).

### 3.5. Overexpression of BraPBL Enhances Resistance

**Figure 5:**
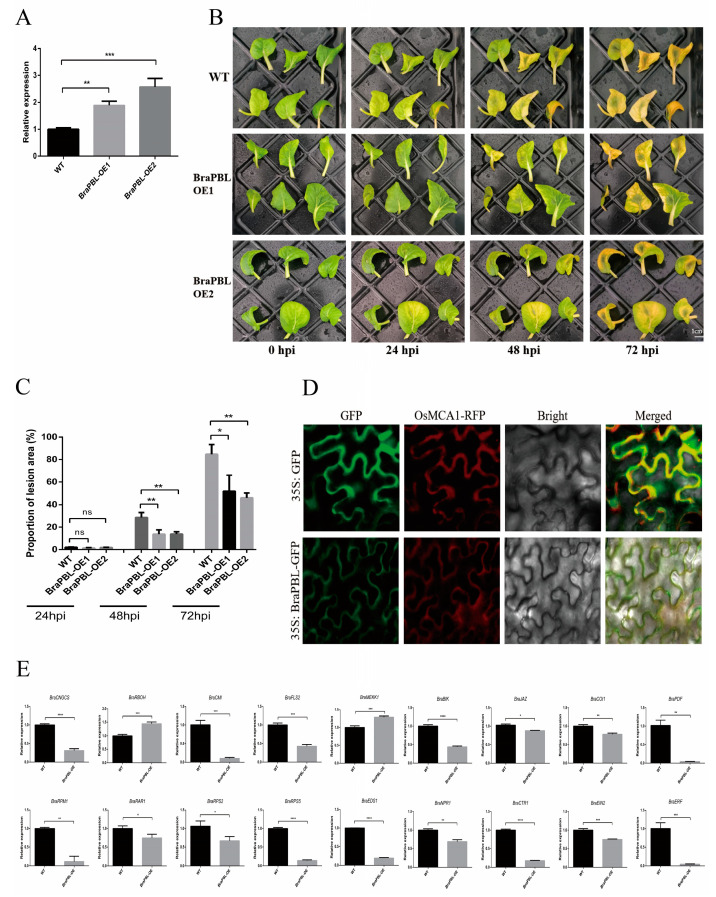
Overexpression of *BraPBL*. (**A**) Expression levels in transgenic Chinese cabbage; (**B**) Disease symptoms of transgenic Chinese cabbage, bar = 1 cm; (**C**) Percentage of lesion area of transgenic Chinese cabbage; (**D**) Subcellular localization of BraPBL in tobacco, with OsMCA1-RFP as a plasma membrane marker; (**E**) Expression of disease defence pathway and hormone signal transduction pathway -related genes in *BraPBL*-overexpression plants. ****, ***, **, *, and ns indicate *p* < 0.0001, *p* < 0.001, *p* < 0.01, *p* < 0.05, and not significant, respectively. Data are presented as means ± standard deviation from three independent biological replicates.

The gene *BraPBL* was overexpressed in disease-susceptible Chinese cabbage line 49caixin. Transgenic lines were confirmed by PCR and expression analysis (Figure 5A, Supplementary Figure S3). Pathogen inoculation assays were then conducted on excised leaves, with statistical evaluation across treatments. At 24 hpi, all treatment groups remained at disease severity level 1. At 48 hpi, the control group exhibited a disease severity level of 7. Leaves inoculated with the pathogen develop brown spots covered with a layer of mould, featuring a yellow halo and distinct necrotic areas. Whereas overexpression lines (*BraPBL*-OE1 and *BraPBL*-OE2) remained substantially less affected. The *BraPBL* overexpression lines exhibited a disease severity level of 5. Leaves develop brown spots with sparse mould growth, featuring a yellow halo. The disease severity in each treatment group further intensified at 72 hpi. The control group exhibited a disease severity level of 9, whereas the *BraPBL* overexpression group demonstrated a severity level of 7 (Figure 5B,C).

Subcellular localization analysis revealed that *BraPBL* is expressed at the plasma membrane (Figure 5D). Expression levels of genes involved in the disease defence pathway and hormone signal transduction pathway were analysed from the *BraPBL*-overexpressing plants. The reactive oxygen species-related gene, *RBOH*, and the signal transduction-related gene, *MEKK1*, showed significantly upregulated expression. The negatively regulated genes, *COI1* and *JAZ*, in the JA pathway were both downregulated. Genes, *EIN2* and *ERF*, in the ET pathway, along with the gene, *NPR1*, in the SA pathway, were downregulated (Figure 5E).

## 4. Discussion

Plant hormones and their associated signalling networks form a central regulatory layer of plant immunity. JA is a key defence hormone that enhances resistance to necrotrophic pathogens but tends to increase susceptibility to biotrophic pathogens [40]. SA mediates plant defence by inducing local defence and systemic acquired resistance. ET generally acts synergistically with JA to promote defence against necrotrophs, while both hormones antagonise SA-mediated responses against biotrophic pathogens. The JA–ET pathways exert coordinated immune outputs through ERF transcription factors, whereas NPR1 and TGA function as core regulators of SA–ET/JA antagonism [41]. Consistent with this framework, 904B Chinese cabbage activated multiple hormonal pathways after infection. The gene *COI-1* in the JA pathway and the gene *CTR1* in the ET pathway were both significantly downregulated. Meanwhile, no differential expression was detected in the genes of the SA pathway. Resistance/susceptibility to *A. brassicicola* appears to be regulated by the interplay between SA-dependent resistance to biotrophic pathogens and JA-dependent resistance to necrotrophic pathogens [42,43]. Similarly, analogous hormonal response strategies are observed in other species. *A. brassicicola* can redirect the defence response towards the biotrophic phase by enhancing the SA response in the susceptible *Brassica juncea*. *Sinapis alba* enhances ABA hormone signalling, which not only counteracts the SA response but also restores the necrotrophic resistance pattern by boosting JA biosynthesis, thereby enhancing disease resistance [44,45]. Although auxin and cytokinins are traditionally associated with growth regulation, both have been implicated in modulating pathogen resistance [46]. A total of five DEGs were detected in the auxin pathway, with only *AUX1* exhibiting upregulation, while the remaining genes all showed downregulation. Both key genes *ARR-A* and *ARR-B* in the cytokinin pathway were downregulated. These patterns indicate that 904B rapidly suppresses the gene in growth-associated hormone pathways early after infection, prioritising defence over development. Research into Chinese cabbage resistance to clubroot disease has also revealed that following pathogen infection, there is a significant reduction in auxin and cytokinin levels, alongside suppression of gene expression in related signalling pathways (*GH3*, *IAA16*, *SAUR32*, *ARR12*, and *LAX2*). This hormonal reprogramming event, in conjunction with defence hormone signals such as JA, SA, and ET, collectively constitutes an effective pathogen defence mechanism [47,48]. However, the specific mechanisms underlying the interactions between these growth-related hormones and disease-defence-related hormones remain to be further elucidated.

In addition to hormones, multiple secondary metabolites have been identified in the disease defence process of Chinese cabbage. Plants produce secondary metabolites as part of their defence mechanism to counteract various biotic stresses. Under conditions of stress, metabolites accumulate to high levels, acting not only as signalling molecules but also enhancing the expression of genes involved in plant defence pathways. Certain secondary metabolites also exert inhibitory effects on pathogens and pests. Moreover, secondary metabolites confer resilience upon plants, enhancing the ability to withstand adverse environmental conditions [49]. Plant secondary metabolites are diverse in variety and, based on the basic structural characteristics, can be categorised into three major classes: terpenoids, phenolics, and nitrogen-containing compounds. Terpenes encompass alcohols, aldehydes, carboxylic acids, ketones, esters, and glycosides. Phenolic compounds encompass flavonoids, simple phenols, and quinones. Nitrogen-containing compounds include alkaloids, cyanogenic glycosides, and non-protein amino acids [50]. Secondary metabolites can serve as mechanical barriers and biochemical defences against pathogen invasion, and also function as signalling molecules in plant disease resistance responses [51,52]. This study revealed that multiple secondary metabolites exhibited differential changes, specifically the sterol compound 4,4-dimethyl-5alpha-cholest-7-en-3beta-ol was significantly upregulated in the treatment group. The 4,4-dimethyl-5alpha-cholest-7-en-3beta-ol is a type of phytosterol. It is an important component of plant cell membranes [53]. Sterol molecules insert into phospholipid bilayers, enhancing membrane rigidity and stability to counteract damage to cells from external environmental fluctuations [54]. The accumulation of this compound may bolster the stress resistance of cell membranes, aiding plants in adapting to environmental pressures [55]. Exogenous sterol treatment enhances plant tolerance to extreme temperatures, salinity, and drought [56,57,58]. This compound also serves as a key intermediate in the plant sterol biosynthesis pathway, participating in the synthesis of downstream active sterols, particularly brassinosterols [59,60]. Brassinosterols are key hormones in the growth and development of Chinese cabbage, regulating cell elongation, division, and stress resistance [61]. Concurrently, we observed upregulation of the *CYCD3* gene within the brassinolide pathway in the transcriptome.

RLCKs constitute a family of serine/threonine kinases localised to the plant plasma membrane, playing a pivotal role in plant immune signal transduction. As key downstream components of plant pattern recognition receptors, RLCKs receive recognition signals from pathogen-associated molecular patterns and activate downstream immune responses through phosphorylation. RLCKs phosphorylate diverse substrates—including NADPH oxidases, G-proteins, and phosphatases—and thereby regulate plant innate immunity, stress adaptation, and hormone responses [62]. Here, RLCK family members in 904B increased in both number and expression after being infected. Subcellular localization confirmed BraPBL at the plasma membrane. Functional analyses further demonstrated that silencing *BraPBL* reduced resistance to black spot disease, whereas overexpression of *BraPBL* enhanced resistance. These results identify *BraPBL* as a black-spot-induced defence gene. Detection of gene expression levels in disease resistance defence pathways and hormone signal transduction pathways in *BraPBL*-overexpressing plants. It was found that the reactive oxygen species-related gene *RBOH* and the signal transduction-related gene *MEKK1* exhibited significantly upregulated expression. Negatively regulating genes, *COI1* and *JAZ*, are both downregulated, indicating that the JA pathway is activated. The genes *EIN2* and *ERF* are both downregulated, indicating that the ET pathway is suppressed. Downregulation of the *NPR1* gene indicates that the SA pathway is inhibited. These findings collectively suggest that the *BraPBL* gene may exert its effects by simultaneously activating the JA pathway and upregulating the reactive oxygen species-related gene, *RBOH*, and the signal transduction-related gene *MEKK1*. RLCKs serve as a crucial bridge linking early pathogen recognition to hormone-mediated defence responses in plant immunity [63]. Upon sensing EF-Tu, a member of the RLCKs family, the *BIK1* gene can translocate from the plasma membrane into the nucleus and phosphorylate WRKY33 and WRKY50/57, thereby regulating the SA and JA signalling pathways [64]. BIK1 regulates ET signalling by interacting with PEPR1. The tomato receptor-like cytoplasmic kinase, SlZARK1, regulates JA accumulation [65,66]. In *Arabidopsis*, *ATPBL1* and its homologue *ATBIK1* act as downstream hubs for multiple PRRs [67], becoming hyperphosphorylated upon PAMP perception and activating Ca^2+^-permeable channels and cyclic nucleotide-gated channels to initiate ROS bursts and stomatal immunity [68]. *BraPBL* may operate through a similar mechanism, although this requires further validation.

## 5. Conclusions

Within 24 hpi, the genes in auxin and cytokinin signalling are broadly suppressed in the Chinese cabbage line 904B. In contrast, the genes in ET and JA signalling are activated. Concurrently, *BraPBL* exhibits increasing expression with prolonged infection. *BraPBL* overexpression enhances resistance to black spot disease, whereas silencing reduces resistance. Collectively, these findings highlight coordinated hormone reprogramming and RLCKs-mediated signalling as key components of the Chinese cabbage defence response.

## Data Availability

The data presented in this study are openly available in the NCBI database (SRR28286398, SRR28286399, SRR28286400, SRR28286402, SRR28286392, SRR28286391).

## Supplementary Materials

The following supporting information can be downloaded at: https://www.mdpi.com/article/10.3390/genes17010115/s1. Figure S1: Correlation between RT-qPCR and DEG expression; Figure S2: Gel plot of different treatment groups in the VIGS experiment; Figure S3: Gel plot of different treatment groups in the overexpression experiment; Table S1: Primer sequences; Table S2: Statistics of transcriptome sequencing data for all samples; Table S3: Expression of transcription factors in different treatment groups; Table S4: Metabolites detected in different treatment groups.

## References

[1] Ma W., Zhang P., Zhao J., Hong Y. (2023). Chinese cabbage: An emerging model for functional genomics in leafy vegetable crops. Trends Plant Sci..

[2] Brazauskienė I., Petraitienė E., Brazauskas G., Semaškienė R. (2011). Medium-term trends in dark leaf and pod spot epidemics in *Brassica napus* and *Brassica rapa* in Lithuania. J. Plant Dis. Prot..

[3] Meena P.D., Meena R.L., Chattopadhyay C., Kumar A. (2004). Identification of critical stage for disease development and biocontrol of Alternaria blight of Indian mustard (*Brassica juncea*). J. Phytopathol..

[4] Köhl J., Van Tongeren C.A.M., Groenenboom-de Haas B.H., Van Hoof R.A., Driessen R., Van Der Heijden L. (2010). Epidemiology of dark leaf spot caused by *Alternaria brassicicola* and *A. brassicae* in organic seed production of cauliflower. Plant Pathol..

[5] Gao J., Liu Y.N., Nan N., Lu B.H., Xia W.Y., Wu X.Y. (2014). *Alternaria brassicicola* causes a leaf spot on isatis indigotica in China. Plant Dis..

[6] Nowicki M., Nowakowska M., Niezgoda A., Kozik E. (2012). Alternaria black spot of crucifers: Symptoms, importance of disease, and perspectives of resistance breeding. J. Fruit. Ornam. Plant Res..

[7] Macioszek V.K., Gapińska M., Zmienko A., Sobczak M., Skoczowski A., Oliwa J., Kononowicz A.K. (2020). Complexity of *Brassica oleracea*–*Alternaria brassicicola* susceptible interaction reveals downregulation of photosynthesis at ultrastructural, transcriptional, and physiological levels. Cells.

[8] Runno-Paurson E., Lääniste P., Nassar H., Hansen M., Eremeev V., Metspalu L., Edesi L., Kännaste A., Niinemets Ü. (2021). Alternaria black spot (*Alternaria brassicae*) infection severity on Cruciferous oilseed crops. Appl. Sci..

[9] Humpherson-Jones F.M., Phelps K. (1989). Climatic factors influencing spore production in *Alternaria brassicae* and *Alternaria brassicicola*. Ann. Appl. Biol..

[10] Jones J.D., Dangl J.L. (2006). The plant immune system. Nature.

[11] Hatsugai N., Igarashi D., Mase K., Lu Y., Tsuda Y., Chakravarthy S., Wei H.L., Foley J.W., Collmer A., Glazebrook J. (2017). A plant effector-triggered immunity signaling sector is inhibited by pattern-triggered immunity. EMBO.

[12] Heath M.C. (2000). Hypersensitive response-related death. Plant Mol. Biol..

[13] Shirasu K., Schulze-Lefert P. (2000). Regulators of cell death in disease resistance. Plant Mol. Biol..

[14] Lorang J.M., Sweat T.A., Wolpert T.J. (2007). Plant disease susceptibility conferred by a “resistance” gene. Proc. Natl. Acad. Sci. USA.

[15] Chisholm S.T., Coaker G., Day B., Staskawicz B.J. (2006). Host-microbe interactions: Shaping the evolution of the plant immune response. Cell.

[16] Nawaz M., Sun J., Shabbir S., Khattak W.A., Ren G., Nie X., Bo Y., Javed Q., Du D., Sonne C. (2023). A review of plants strategies to resist biotic and abiotic environmental stressors. Sci. Total Environ..

[17] Duhan L., Pasrija R. (2025). Unveiling exogenous potential of phytohormones as sustainable arsenals against plant pathogens: Molecular signaling and crosstalk insights. Mol. Biol. Rep..

[18] Das D., Kashtoh H., Panda J., Rustagi S., Mohanta Y.K., Singh N., Baek K.H. (2025). From hormones to harvests: A pathway to strengthening plant resilience for achieving sustainable development goals. Plants.

[19] Schaller H. (2003). The role of sterols in plant growth and development. Prog. Lipid Res..

[20] Rogowska A., Szakiel A. (2020). The role of sterols in plant response to abiotic stress. Phytochem. Rev..

[21] Zhu J.K. (2016). Abiotic stress signaling and responses in plants. Cell.

[22] Zipfel C., Oldroyd G.E. (2017). Plant signalling in symbiosis and immunity. Nature.

[23] Lin W., Ma X., Shan L., He P. (2013). Big roles of small kinases: The complex functions of receptor-like cytoplasmic kinases in plant immunity and development. J. Integr. Plant Biol..

[24] Lehti-Shiu M.D., Zou C., Hanada K., Shiu S.H. (2009). Evolutionary history and stress regulation of plant receptor-like kinase/pelle genes. Plant Physiol..

[25] Vij S., Giri J., Dansana P.K., Kapoor S., Tyagi A.K. (2008). The receptor-like cytoplasmic kinase (OsRLCK) gene family in rice: Organization, phylogenetic relationship, and expression during development and stress. Mol. Plant.

[26] Liang X., Zhang J. (2022). Regulation of plant responses to biotic and abiotic stress by receptor-like cytoplasmic kinases. Stress. Biol..

[27] Hailemariam S., Liao C.J., Mengiste T. (2024). Receptor-like cytoplasmic kinases: Orchestrating plant cellular communication. Trends Plant Sci..

[28] Liang X., Zhou J.M. (2018). Receptor-like cytoplasmic kinases: Central players in plant receptor kinase-mediated signaling. Annu. Rev. Plant Biol..

[29] Yamaguchi K., Yamada K., Kawasaki T. (2013). Receptor-like cytoplasmic kinases are pivotal components in pattern recognition receptor-mediated signaling in plant immunity. Plant Signal Behav..

[30] Yan W., Zhang H., Fan W., Liu X., Huang Z., Wang Y., Wang C., Zhang B. (2025). Chromosome-level genome assembly of Chinese cabbage J405 reveals underlying high resistance to *Alternaria brassicicola*-induced black spot disease. Plant Stress..

[31] Chen S., Zhou Y., Chen Y., Gu J. (2018). Fastp: An ultra-fast all-in-one FASTQ preprocessor. Bioinformatics.

[32] Kim D., Paggi J.M., Park C. (2019). Graph-based genome alignment and genotyping with HISAT2 and HISAT-genotype. Nat. Biotechnol..

[33] Pertea M., Pertea G.M., Antonescu C.M., Chang T.C., Mendell J.T., Salzberg S.L. (2015). StringTie enables improved reconstruction of a transcriptome from RNA-seq reads. Nat. Biotechnol..

[34] Love M.I., Huber W., Anders S. (2014). Moderated estimation of fold change and dispersion for RNA-seq data with DESeq2. Genome Biol..

[35] Boccard J., Rutledge Douglas N.A. (2013). Consensus orthogonal partial least squares discriminant analysis (OPLS-DA) strategy for multiblock Omics data fusion. Anal. Chim. Acta.

[36] Chong J., Xia J. (2018). MetaboAnalystR: An R package for flexible and reproducible analysis of metabolomics data. Bioinformatics.

[37] Livak K.J., Schmittgen T.D. (2001). Analysis of relative gene expression data using real-time quantitative PCR and the 2^−∆∆Ct^ method. Methods.

[38] Xiong X., Liu W., Jiang J., Xu L., Huang L., Cao J. (2019). Efficient genome editing of *Brassica campestris* based on the CRISPR/Cas9 system. Mol. Genet. Genom..

[39] Kurusu T., Nishikawa D., Yamazaki Y., Gotoh M., Nakano M., Hamada H., Yamanaka T., Iida K., Nakagawa Y., Saji H. (2012). Plasma membrane protein OsMCA1 is involved in regulation of hypo-osmotic shock-induced Ca^2+^ influx and modulates generation of reactive oxygen species in cultured rice cells. BMC Plant Biol..

[40] Zhang N., Zhou S., Yang D., Fan Z. (2020). Revealing shared and distinct genes responding to JA and SA signaling in Arabidopsis by meta-analysis. Front. Plant Sci..

[41] Caarls L., Pieterse C.M., Van-Wees S.C. (2015). How salicylic acid takes transcriptional control over jasmonic acid signaling. Front. Plant Sci..

[42] Spoel S.H., Johnson J.S., Dong X. (2007). Regulation of tradeoffs between plant defenses against pathogens with different lifestyles. Proc. Natl. Acad. Sci. USA.

[43] Flors V., Ton J., Van-Doorn R., Jakab G., García-Agustín P., Mauch-Mani B. (2008). Interplay between JA, SA and ABA signalling during basal and induced resistance against *Pseudomonas syringae* and *Alternaria brassicicola*. Plant J..

[44] Mazumder M., Das S., Saha U., Chatterjee M., Bannerjee K., Basu D. (2013). Salicylic acid-mediated establishment of the compatibility between *Alternaria brassicicola* and *Brassica juncea* is mitigated by abscisic acid in *Sinapis alba*. Plant Physiol. Biochem..

[45] De A., Maity A., Mazumder M., Mondal B., Mukherjee A., Ghosh S., Ray P., Polley S., Dastidar S.G., Basu D. (2021). Overexpression of LYK4, a lysin motif receptor with non-functional kinase domain, enhances tolerance to *Alternaria brassicicola* and increases trichome density in *Brassica juncea*. Plant Sci..

[46] Ding X., Cao Y., Huang L., Zhao J., Xu C., Li X., Wang S. (2008). Activation of the indole-3-acetic acid-amido synthetase GH3-8 suppresses expansin expression and promotes salicylate- and jasmonate-independent basal immunity in rice. Plant Cell.

[47] Wei X., Zhang Y., Zhao Y., Xie Z., Hossain M.R., Yang S., Shi G., Lv Y., Wang Z., Tian B. (2021). Root transcriptome and metabolome profiling reveal key phytohormone-related genes and pathways involved clubroot resistance in *Brassica rapa* L. Front. Plant Sci..

[48] Ludwig-Müller J. (2014). Auxin homeostasis, signaling, and interaction with other growth hormones during the clubroot disease of Brassicaceae. Plant Signal Behav..

[49] Erb M., Kliebenstein D.J. (2020). Plant secondary metabolites as defenses, regulators, and primary metabolites: The blurred functional trichotomy. Plant Physiol..

[50] Zaynab M., Fatima M., Abbas S., Sharif Y., Umair M., Zafar M.H., Bahadar K. (2018). Role of secondary metabolites in plant defense against pathogens. Microb. Pathog..

[51] Tang H., Wang Q., Xie H., Li W. (2024). The function of secondary metabolites in resisting stresses in horticultural plants. Fruit Res..

[52] Ahmed E., Arshad M., Khan M.Z., Amjad M.S., Sadaf H.M., Riaz I., Sabir S., Ahmad N., Sabaoon (2017). Secondary metabolites and their multidimensional prospective in plant life. J. Pharmacogn. Phytochem..

[53] Rahier A. (2011). Dissecting the sterol C-4 demethylation process in higher plants. From structures and genes to catalytic mechanism. Steroids.

[54] Du Y., Fu X., Chu Y., Wu P., Liu Y., Ma L., Tian H., Zhu B. (2022). Biosynthesis and the roles of plant sterols in development and stress responses. Int. J. Mol. Sci..

[55] Vogel P., Persson S., Moreno-Pescador G., Noack L.C. (2025). Sterols in plant biology-advances in studying membrane dynamics. Cell Surf..

[56] Grosjean K., Mongrand S., Beney L., Simon-Plas F., Gerbeau-Pissot P. (2015). Differential effect of plant lipids on membrane organization: Specificities of phytosphingolipids and phytosterols. J. Biol. Chem..

[57] Abu-Muriefah S.S. (2015). Effect of sitosterol on growth, metabolism and protein pattern of pepper (*Capsicum annuum* L.) plants grown under salt stress conditions. Intl. J. Agric. Crop Sci..

[58] Elkeilsh A., Awad Y.M., Soliman M.H., Abu-Elsaoud A., Abdelhamid M.T., El-Metwally I.M. (2019). Exogenous application of β-sitosterol mediated growth and yield improvement in water-stressed wheat (*Triticum aestivum*) involves up-regulated antioxidant system. J. Plant Res..

[59] Yin X., Liu J., Kou C., Lu J., Zhang H., Song W., Li Y., Xue Z., Hua X. (2023). Deciphering the network of cholesterol biosynthesis in paris polyphylla laid a base for efficient diosgenin production in plant chassis. Metab. Eng..

[60] Bajguz A., Chmur M., Gruszka D. (2020). Comprehensive overview of the brassinosteroid biosynthesis pathways: Substrates, products, inhibitors, and connections. Front. Plant Sci..

[61] Liu Y., Huang X., Li M., He P., Zhang Y. (2016). Loss-of-function of Arabidopsis receptor-like kinase BIR1 activates cell death and defense responses mediated by BAK1 and SOBIR1. New Phytol..

[62] Wang J., Bai L., Xu Y., Zheng X., Shan W., Shi X., Ma S., Fan J. (2025). Receptor-like cytoplasmic kinases mediated signaling in plant immunity: Convergence and divergence. Stress. Biol..

[63] Lal N.K., Nagalakshmi U., Hurlburt N.K., Flores R., Bak A., Sone P., Ma X., Song G., Walley J., Shan L. (2018). The receptor-like cytoplasmic kinase BIK1 localizes to the nucleus and regulates defense hormone expression during plant innate immunity. Cell Host Microbe.

[64] Veronese P., Nakagami H., Bluhm B., Abuqamar S., Chen X., Salmeron J., Dietrich R.A., Hirt H., Mengiste T. (2006). The membrane-anchored BOTRYTIS-INDUCED KINASE1 plays distinct roles in Arabidopsis resistance to necrotrophic and biotrophic pathogens. Plant Cell.

[65] Liu Z., Wu Y., Yang F., Zhang Y., Chen S., Xie Q., Tian X., Zhou J.M. (2013). BIK1 interacts with PEPRs to mediate ethylene-induced immunity. Proc. Natl. Acad. Sci. USA.

[66] Sun Z., Zang Y., Zhou L., Song Y., Chen D., Zhang Q., Liu C., Yi Y., Zhu B., Fu D. (2021). Atomato receptor-like cytoplasmic kinase SlZRK1, acts as a negative regulator in wound-induced jasmonic acid accumulation insect resistance. J. Exp. Bot..

[67] Zhang J., Li W., Xiang T., Liu Z., Laluk K., Ding X., Zou Y., Gao M., Zhang X., Chen S. (2010). Receptor-like cytoplasmic kinases integrate signaling from multiple plant immune receptors and are targeted by a *Pseudomonas syringae* effector. Cell Host Microbe.

[68] Bai J., Zhou Y., Sun J., Chen K., Han Y., Wang R., Zou Y., Du M., Lu D. (2023). BIK1 protein homeostasis is maintained by the interplay of different ubiquitin ligases in immune signaling. Nat. Commun..

