# Supplementary material for: Multi-Omics Analysis Identifies the Key Defence Pathways in Chinese Cabbage Responding to Black Spot Disease
Source: Genes (Basel). 2026 Jan 21;17(1):115. doi: 10.3390/genes17010115 (PMC12840714; doi:10.3390/genes17010115)
Supplement: Supplementary file 1 [file genes-17-00115-s001.zip › Supplymentary Figure.pdf]

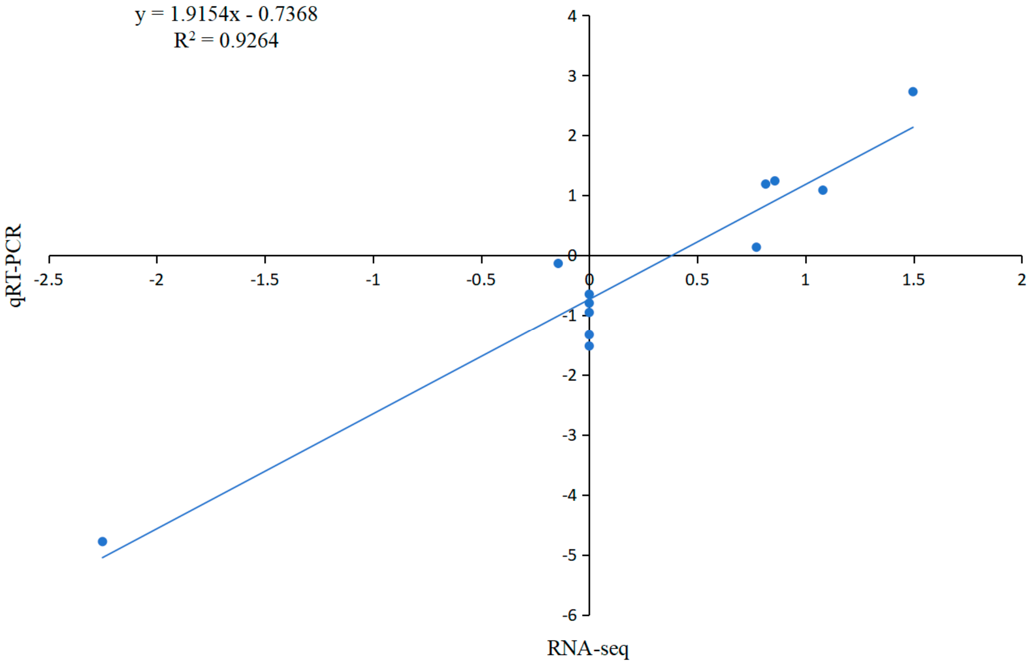

Figure S1. Correlation between RT-qPCR and DEG expression.

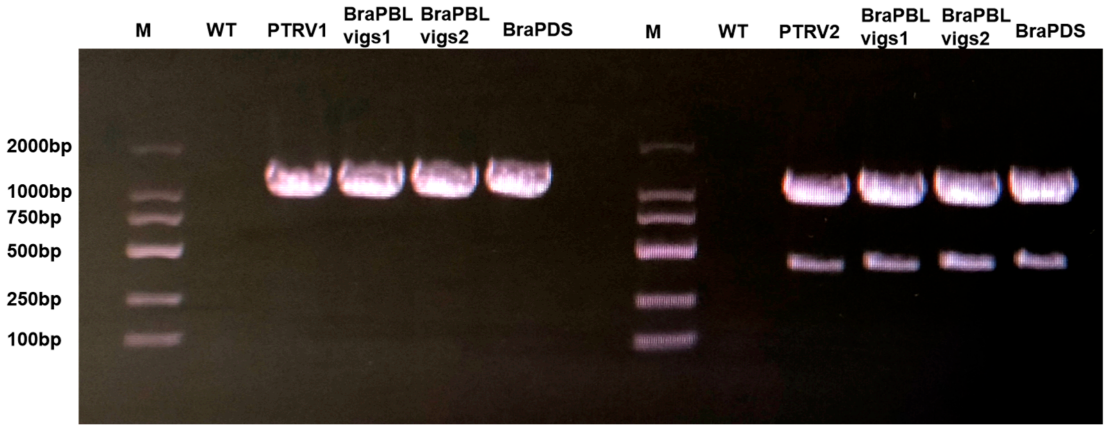

Figure S2. PCR gel plot of different treatment groups in the VIGS experiment.

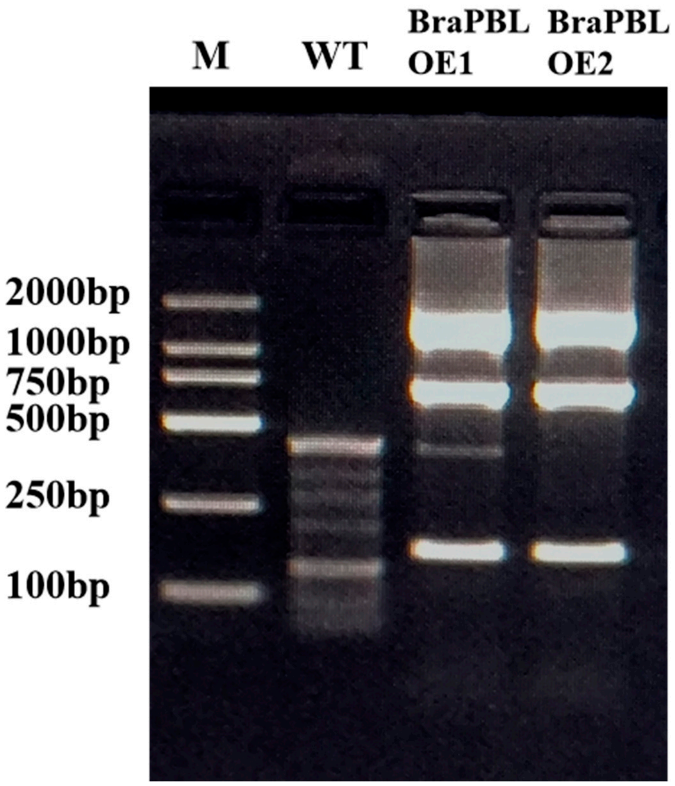

Figure S3. PCR gel plot of different treatment groups in the overexpression experiment.
